# Nalbuphine as an Intrathecal Adjuvant to 0.5% Hyperbaric Bupivacaine in Two Different Doses for Postoperative Analgesia After Abdominal Hysterectomy: A Prospective, Randomized, Double-Blind Control Study

**DOI:** 10.7759/cureus.25044

**Published:** 2022-05-16

**Authors:** Mahnaz S Shah, Tahir Masoodi, Sana Y Hussain, Dhruv Jain

**Affiliations:** 1 Anaesthesiology, Hamdard Institute of Medical Sciences and Research, New Delhi, IND; 2 Anaesthesiology, Pain Medicine and Critical Care, All India Institute of Medical Sciences, New Delhi, IND

**Keywords:** sensory block, local anesthetic adjuvants, anesthesia spinal, intrathecal, nalbuphine

## Abstract

Introduction: Adding adjuvant drugs to intrathecal local anesthetics improves the quality and duration of the sensory blockade and prolongs postoperative analgesia. Intrathecal opioids are synergistic with local anesthetics, thereby intensifying the sensory block without increasing the sympathetic block. This study was designed to comparatively evaluate the two different dosages of nalbuphine as intrathecal adjuvants on subarachnoid block (SAB) characteristics of 0.5% hyperbaric bupivacaine.

Methods: A randomized, triple arm study was conducted on 60 adult female patients with American Society of Anesthesiologists physical status I and II, aged 30-60 years, scheduled for total abdominal hysterectomy under SAB. Patients were randomized into three groups: group I received 15 mg of 0.5% hyperbaric bupivacaine, group II received 15 mg of 0.5% hyperbaric bupivacaine with 1.6 mg of nalbuphine, and group III received 15 mg of 0.5% hyperbaric bupivacaine with 2.4 mg of nalbuphine. The primary outcome was the duration of analgesia, while secondary outcomes included onset, duration of sensory and motor block, maximum cephalic extension, and two dermatome segment regressions.

Results: The onset time of the sensory block was 3.2 ± 1.0 minutes, 3.5 ± 1.6 minutes, and 3.1 ± 1.1 minutes in groups I, II, and III, respectively. The onset time of the motor block was 8.5 ± 1.0 minutes, 8.5 ± 1.1 minutes, and 8.2 ± 1.1 minutes in groups I, II, and III, respectively. The onset of sensory and motor blocks was comparable among the three groups with no statistically significant difference (p > 0.05). The total duration of analgesia was 117.8 ± 23.3 minutes, 166.8 ± 27.8 minutes, and 181.8 ± 25.9 minutes in groups I, II, and III, respectively, with a statistically significant difference. Few incidences of manageable hypotension, but no incidences of bradycardia or respiratory insufficiency, occurred. Five patients of the control group shivered, which was managed well by tramadol 50 mg and ondansetron 4 mg. No patient suffered from pruritus, sedation, respiratory depression, nausea, and vomiting.

Conclusion: The study concluded that intrathecal nalbuphine in a 1.6 mg dose is an effective adjuvant to 0.5% hyperbaric bupivacaine for SAB. It potentiated the SAB characteristics and enhanced the duration of analgesia with no effect on respiration. Nalbuphine in a dose of 2.4 mg did not offer any added advantage.

## Introduction

Subarachnoid blockade is a common anesthetic technique for lower abdominal and lower limb surgeries. Adding adjuvant drugs to intrathecal local anesthetics improves the quality and duration of the sensory blockade and prolongs postoperative analgesia. Intrathecal opioids are synergistic with local anesthetics, thereby intensifying the sensory block without increasing sympathetic block [1], and are the most commonly utilized spinal adjuvants to prolong postsurgical analgesia [2].

Nalbuphine is a synthetic highly lipid-soluble opioid analgesic and possesses an agonist action at the κ-opioid receptor and antagonist action at the μ-opioid receptor to provide reasonably potent analgesia of visceral nociception [3]. It is used in almost all types of general and regional anesthetic techniques. Nalbuphine binds to kappa receptors distributed in the spinal cord and brain to produce analgesia. When used as an adjuvant to hyperbaric bupivacaine, it also improves the quality of perioperative analgesia with fewer side effects [4]. It is a mixed synthetic agonist-antagonist, which attenuates the μ-opioid effects and enhances the κ-opioid effects [5].

Nalbuphine, if given systemically, has a reduced incidence of respiratory depression and has been used to antagonize the side effects of spinal opiates [6]. Intrathecal nalbuphine produces lesser adverse effects like pruritus, nausea, and vomiting when compared to intrathecal morphine [7] and does not cause any significant hemodynamic or respiratory complications [8].

This study was designed to comparatively evaluate the two different dosages of nalbuphine as intrathecal adjuvants on subarachnoid characteristics of 0.5% hyperbaric bupivacaine. The primary outcome was the duration of analgesia. Secondary outcomes included maximum cephalic extension, two dermatome segment regression, duration of motor block, hemodynamic variations, shivering, pruritus, sedation, nausea/vomiting, and respiratory depression.

## Materials and methods

Selection of patients

After approval from the institutional ethical committee, 60 adult consenting female patients with American Society of Anesthesiologists (ASA) physical status I and II, aged 30-60 years, scheduled for total abdominal hysterectomy under the subarachnoid block (SAB) were enrolled for the present study after taking written informed consent. All selected patients underwent pre-anesthetic evaluation for airway assessment and systemic examination.

Exclusion criteria

The exclusion criteria included: (1) patients with a history of pre-existing cardiac or pulmonary disease, renal or hepatic derangements, and metabolic or neurological disorders; (2) deformity of the spinal column or cutaneous infection at the lumbar puncture site; (3) bleeding or coagulation disorder; (4) known hypersensitivity or allergy to local anesthetics/opioids; and (5) uncooperative patients or refusal to the technique.

Group allocation

All patients were randomized into three equal groups of 20 patients each by a computer-generated random number table. Group I received 15 mg of 0.5% hyperbaric bupivacaine alone. Group II received 15 mg of 0.5% hyperbaric bupivacaine with 1.6 mg of nalbuphine. Group III received 15 mg of 0.5% hyperbaric bupivacaine with 2.4 mg of nalbuphine.

Method of blinding

The total volume of the study drug solution was kept at 3.5 mL by normal saline. The drug solution was prepared by another resident anesthesiologist who was not further involved in data collection. The anesthesiologist who performed the SAB was also blinded to the study group allocation.

Subarachnoid blockade technique

All selected patients were given 0.25 mg alprazolam tablet orally on the night before surgery and six-hour fasting was ensured. They were pre-hydrated with 10 ml/kg Ringer lactate solution 30 minutes before initiation of SAB to replenish the overnight fasting. Monitoring for heart rate, systemic blood pressure, electrocardiography, and peripheral pulse oximetry was commenced by using a multipara monitor. Under aseptic conditions, the intrathecal injection of 3.5 ml of study drug solution was given in a sitting position at the L2-3 or L3-4 interspaces through a midline approach using a 25 G Quincke’s spinal needle with the bevel pointing upwards. Immediately after, patients were made to lie supine and the table was positioned to provide a 10° head down tilt to achieve the required level of surgical anesthesia. All patients were supplemented with oxygen with a face mask at 5 liter/minute.

The sensory block characteristics were assessed by the pinprick method for the onset time at the T10 dermatome, maximum cephalic extension, and time for two dermatome regressions. The motor block was assessed by the modified Bromage scale at two-minute intervals until a complete motor block occurred and then every 15 minutes after the completion of surgery. Motor blockade was assessed for the time taken to achieve complete motor blockade of Bromage scale score of 3 and the time taken to complete regression of motor blockade. Modified Bromage scale (0-3) was used as follows: 0 = full movement, no power impairment, and able to raise straight leg; 1 = unable to raise extended leg at the hip but able to flex knee; 2 = unable to flex the knee but able to move ankle joint; and 3 = unable to move hip, knee, or ankle (no motor activity).

Intraoperatively, patients were observed every five minutes for hemodynamic changes in systemic blood pressure (hypotension: systolic blood pressure < 100 mmHg) and heart rate (bradycardia: heart rate < 60 beats/min), and changes in respiratory pattern (respiratory rate and oxygen saturation (SpO2)). Postoperatively, the total duration of analgesia was noted from the time of intrathecal injection to the first requirement of analgesic (on patient demand). Injection ketorolac 30 mg intravenous was administered as rescue analgesia. Blood pressure and heart rate were monitored postoperatively every 15 minutes until the first request for analgesia. Any signs/symptoms of shivering, pruritus, nausea, vomiting, or any other untoward incident were noted. The patients were followed up for six hours after the surgery. If any complications occurred, patients were managed according to a clinical protocol.

Statistical analysis

The sample size was decided to detect at least a clinically significant difference of 30 minutes in the mean duration of motor block and postoperative analgesia among the groups for type 1 error of 0.05% with a power of 80% and 95% confidence limit. Assuming a 5% dropout rate, the final sample size was set at 60 patients. Statistical analysis was done using Statgraphics Centurion software, version 16 (Statgraphics Technologies, Inc., The Plains, VA). Data were compared using Student's t-test, Fisher’s exact test, and analysis of variance (ANOVA). P-value < 0.05 was considered to indicate statistical significance.

## Results

A total of 60 patients were recruited and all patients completed the study. Twenty patients in each group were analyzed. All three groups were comparable with respect to age, weight, ASA physical status, and duration of surgery with no statistically significant difference (Table 1).

**Table 1:** Baseline and demographic characteristics ASA: American Society of Anesthesiologists.

Variable	Group I (control), N = 30	Group II (1.6 mg nalbuphine), N = 30	Group III ( 2.4 mg nalbuphine), N = 30	P-value
ASA physical status grade (I/II)	16/4	18/2	17/3	0.68
Age (years)	40.8 ± 4.7	43.3 ± 6.0	42.5 ± 5.5	0.34
Weight (kilogram)	59.5 ± 5.5	61.2 ± 6.6	62.6 ± 7.2	0.33
Duration of surgery (minutes)	105.9 ± 8.6	106.7 ± 10.8	107.4 ± 13.4	0.91

The primary and secondary outcome measures are shown in Table 2. The time for two-segment regression was significantly higher in groups II and III compared to group I (p < 0.001), while there was no significant difference between groups II and III. The total duration of analgesia was 117.8 ± 23.3 minutes, 166.8 ± 27.8 minutes, and 181.8 ± 25.9 minutes in groups I, II, and III, respectively, with a statistically significant difference between control and nalbuphine groups (p < 0.001), with no difference amongst groups II and III.

**Table 2:** Characteristics of sensory and motor block

Parameters	Group I	Group II	Group III	P-value
Onset of sensory block (minutes)	3.5 ± 1	3.4 ± 1.6	3.1 ± 1.1	0.58
Onset of motor block (minutes)	8.5 ± 1.0	8.5 ± 1.1	8.2 ± 1.1	0.6
Time for two-segment regression (minutes)	82.4 ± 23.1	117.2 ± 21.1	132.7 ± 23.9	<0.001
Total duration of analgesia (minutes)	117.8 ± 23.3	166.8 ± 25.9	181.8 ± 27.8	<0.001
Total duration of motor block (minutes)	116.6 ± 11	123.2 ± 13.6	123.6 ± 10.5	0.11

Few incidences of manageable hypotension, but no incidences of bradycardia or respiratory insufficiency, occurred. Hypotension was managed by administering 250 ml of Ringer lactate solution as a bolus. Vasopressors for managing hypotension were not required in any patient. Hemodynamic parameters were comparable amongst all the three groups. Five patients of the control group shivered, which was managed well by tramadol 50 mg and ondansetron 4 mg. No patient suffered from pruritus, sedation, respiratory depression, nausea, and vomiting.

## Discussion

In this study, intrathecal nalbuphine potentiated the SAB characteristics and enhanced the duration of effective analgesia (time from injection to visual analog scale (VAS) score < 3) without affecting the hemodynamic profile of patients. The duration of analgesia was significantly larger by adding nalbuphine as an adjuvant with both the doses, i.e., 1.6 and 2.4 mg, being similar. There were no incidences of pruritus, sedation, respiratory depression, nausea, or vomiting, as these symptoms occur due to the action of the µ receptor.

Previous studies [8,9] have shown that nalbuphine increases the duration of analgesia in patients undergoing surgeries under SAB and there was no increase in the duration of motor block. In our study too, the addition of nalbuphine significantly increased the duration of analgesia without affecting the time duration of motor block. Nalbuphine has been compared to other commonly used spinal adjuvants like morphine and fentanyl. In a study by Culebras et al. on patients undergoing cesarean section, morphine resulted in a longer duration of analgesia than nalbuphine as an intrathecal adjuvant, but with increased side effects like pruritus, nausea, and vomiting [7]. Similar results were seen in another study in patients undergoing hip surgeries [10].

Nalbuphine has been compared to fentanyl in spinal anesthesia in a study by Bindra et al. [11]. It was found that nalbuphine produced longer analgesia than fentanyl in cesarean section, while in a study by Gomaa et al. [12], both fentanyl and nalbuphine produced similar analgesia. However, a study by Prabhakaraiah et al. [13] showed that fentanyl produced lower pain scores postoperatively in lower abdominal surgeries than nalbuphine. In another study by Gupta et al. [3], nalbuphine in a dose of 2 mg was superior to fentanyl for lower limb orthopedic surgeries. Thus, the evidence for comparison between nalbuphine and fentanyl is mixed with a recent meta-analysis showing no difference in duration of analgesia and sensory and motor block [14]. However, their results indicated a lower incidence of hypotension, pruritus, and shivering in the nalbuphine group as compared to opioids.

The optimal dose of nalbuphine required for spinal anesthesia has been studied in various trials. Mukherjee et al. compared doses of 0.2, 0.4, and 0.8 mg of intrathecal nalbuphine and concluded that a dose of 0.4 mg provides adequate analgesia without side effects [9]. Borah et al. studied doses of 0.4, 0.8, and 1.6 mg of nalbuphine and found out that both 0.4 and 0.8 mg provide good quality analgesia, while a dose of 1.6 mg produced adverse effects like nausea, vomiting, bradycardia, and hypotension [15]. A study by Ahmed et al. compared doses of 0.8, 1.6, and 2.4 mg of intrathecal nalbuphine and found that a dose of 1.6 mg was most appropriate for abdominal hysterectomy [16]. This corroborates with our study where a dose of 1.6 mg produced similar analgesia as 2.4 mg without any side effects. Though we used higher doses of intrathecal nalbuphine compared to other studies, we did not observe adverse effects like pruritus, nausea, vomiting, or respiratory depression. Various studies of intrathecal nalbuphine have used doses ranging from 0.4 to 2.4 mg and most have shown peak analgesic effects with minimal side effects at doses from 0.8 to 1.6 mg [14]. Thus, the ceiling effect might be seen at doses of 1.6 mg or lesser, and the dose should be kept within this range.

Our results show that nalbuphine can be a useful adjuvant in spinal anesthesia in patients undergoing abdominal hysterectomies. It significantly prolonged the analgesia without increasing the duration of motor block or producing any adverse effects. This can also be useful in daycare surgeries as patients can ambulate early without experiencing pain or side effects. However, this needs to be validated by measuring pain scores during mobilization in future studies. In centers where fentanyl may not be available due to licensing issues, nalbuphine can prove to be a valuable adjunct.

There were a few limitations in our study. A lower dose of intrathecal nalbuphine could also have been studied in our trial. Though no side effects were observed in any of the doses, a lower dose of 0.8 or 0.4 mg could have been compared. Postoperative pain scores were not measured in our study. Follow-up for 24 hours would have provided us with the data on the analgesic efficacy of nalbuphine for the postoperative period.

## Conclusions

The study concluded that intrathecal nalbuphine in a dose of 1.6 mg is an effective adjuvant to 0.5% hyperbaric bupivacaine in SAB for total abdominal hysterectomies. It potentiated the SAB characteristics and enhanced the duration of analgesia with no effect on respiration. Nalbuphine in a dose of 2.4 mg did not offer any added advantage.
